# Dissemination of Patient Priorities Care: Strategies for Transforming Clinical Care Throughout VHA

**DOI:** 10.1093/geroni/igaf122.483

**Published:** 2025-12-31

**Authors:** Katherine Ritchey, Lea Kiefer, Blythe Chandler, Anne Wang

**Affiliations:** Department of Veterans Affairs, Seattle, Washington, United States; Michael E. DeBakey VA Medical Center, Houston, Texas, United States; Michael E. DeBakey VA Medical Center, Houston, Texas, United States; Michael E. DeBakey VA Medical Center, Houston, Texas, United States

## Abstract

Patient Priorities Care (PPC) is an Age-Friendly approach that aligns healthcare with patient values and priorities, especially for persons with multiple chronic conditions (MCC). It is the preferred approach to elicit and act on ‘what matters’ in Age-Friendly Health Systems (AFHS). Though PPC has been shown to be an evidenced based approach that reduces unwanted care and increases the receipt of supportive care in persons with MCC, large scale health system dissemination had been lacking. Therefore, we used a multi-prong approach to promote clinical integration of PPC VHA wide: establish national VHA partnerships; create integrated electronic health record (EHR) templates and tools; standardize training resources; foster a community of practice. In 2020, the Office of Geriatrics and Extended Care designated PPC as one of four Mentored Partner Programs (MPP). This collaborative learning program provides funding and guidance to frontline staff interested in PPC integration. Additional partnerships with VA Whole Health and AFHS leadership teams have further promoted PPC through AFHS action communities and Whole Health clinical champions. Creation of PPC talent management system training videos provided low-barrier access to most health professionals with professional accreditation. Finally, development of VA EHR tools and implementation website offered frontline staff frameworks and resources for PPC integration. Through these efforts, it is now practiced in a variety of clinical settings in all 170 VHA facilities touching over 10,000 veterans monthly. Lessons learned from our experience can be applicable for other health systems interested in person-centered care transformation, especially in populations with MCC.

